# Cyanobacterial Harmful Algal Blooms in Aquatic Ecosystems: A Comprehensive Outlook on Current and Emerging Mitigation and Control Approaches

**DOI:** 10.3390/microorganisms9071472

**Published:** 2021-07-09

**Authors:** Assaf Sukenik, Aaron Kaplan

**Affiliations:** 1 The Yigal Allon Kinneret Limnological Laboratory, Israel Oceanographic and Limnological Research, P.O. Box 447, Migdal 14950, Israel; 2 Department of Plant and Environmental Sciences, Edmond J. Safra Campus, The Hebrew University of Jerusalem, Givat Ram, Jerusalem 9190401, Israel; aaron.kaplan@mail.huji.ac.il

**Keywords:** cyanobacteria, harmful bloom, bloom prevention, oxidative stress, water treatment

## Abstract

An intensification of toxic cyanobacteria blooms has occurred over the last three decades, severely affecting coastal and lake water quality in many parts of the world. Extensive research is being conducted in an attempt to gain a better understanding of the driving forces that alter the ecological balance in water bodies and of the biological role of the secondary metabolites, toxins included, produced by the cyanobacteria. In the long-term, such knowledge may help to develop the needed procedures to restore the phytoplankton community to the pre-toxic blooms era. In the short-term, the mission of the scientific community is to develop novel approaches to mitigate the blooms and thereby restore the ability of affected communities to enjoy coastal and lake waters. Here, we critically review some of the recently proposed, currently leading, and potentially emerging mitigation approaches in-lake novel methodologies and applications relevant to drinking-water treatment.

## 1. Introduction

Cyanobacteria (also known as Cyanophyta, Cyanoprokaryota, Chloroxybacteria, and blue-green algae) constitute the largest, most diverse, and most widely distributed group of photosynthetic organisms. They were the first to perform oxygenic photosynthesis, splitting the water molecule and providing O_2_ to the atmosphere. Accordingly, they played a major role in many biogeochemical processes that revolutionary impacted the biosphere [1,2,3], including nutrient availability and the development of heterotrophs and O_2_-consuming organisms. Presently, cyanobacteria are important primary producers contributing to the plankton, benthos, epiphyton, and epilithon in various aquatic and terrestrial ecosystems. Over the last few decades, they have become mostly known for their notorious blooms in various water bodies. The bloom-forming genera *Microcystis*, *Anabaena* (*Dolichospermum*), *Aphanizomenon*, *Cylindrospermopsis*, and *Lyngbya* often dominate the spring–fall assemblages in water bodies. This is being attributed to global warming and anthropogenic activities leading to eutrophication.

Cyanobacteria proliferate under favorable conditions of nutrient abundance, warm water temperature, calm weather conditions, and the presence of light, often to the extent of forming a bloom. The ability of many toxic strains to optimize their location in the water column is a meaningful ecological advantage. This is accomplished through buoyancy regulation, which is determined by the balance between gas vesicles inflation and the ballast [4]. Consequently, many species of planktonic cyanobacteria accumulate at the surface, particularly in the morning after consuming a significant portion of their reserve carbohydrates during the dark, and form floating scums. This surface accumulation provide the cyanobacteria with better access to CO_2_ and light while, at the same time, shading the water column below. In addition, allelopathy and competitive exclusion support cyanobacteria blooms and the domination of the planktonic algal assemblage [5]. The proliferation of toxic cyanobacteria is further augmented by low grazing pressure. Blooms of cyanobacteria are undesirable due to the accumulation and rotting of stagnant biomass, resulting in taste and odor problems. More importantly, as many species produce an array of toxins, cyanobacteria blooms constitute a serious health hazard referred to as cyanobacterial harmful algal blooms (cyanoHABs). This has attracted the attention of both regional and national water and nature authorities, as well as the general public affected by cyanoHABs.

**Figure 1:**
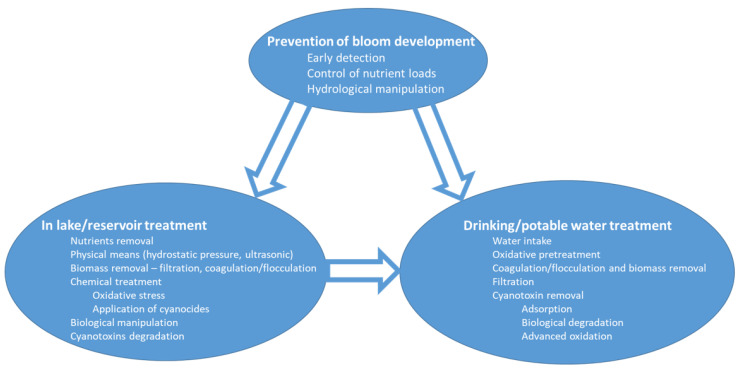
Schematic presentation of strategies and techniques implemented to prevent the development of cyanoHAB and to mitigate potential impacts on aquatic ecosystem and potable water.

Though cyanoHABs and their environmental, health, and social consequences have been reviewed during the last three decades and recently updated [6,7,8], it is imperative to critically review current and emerging approaches to mitigate and control cyanoHABs, their ecological impact, and their effect on consumers via drinking water or recreational activities. Here, we focus on three domains that cover the main levels of cyanoHAB treatment and control: the prevention of bloom development, in lake/reservoir treatment, and in drinking/potable water treatment (Figure 1). We do not wish to provide a comprehensive review for each component; instead, we critically evaluate the methodologies currently proposed to mitigate, eliminate, and control toxic cyanobacteria blooms, as well as possible future developments.

## 2. Prevention of Bloom Development

### 2.1. Early Detection

Naturally, reliable and sensitive approaches capable of the early detection of cyanoHABs are vital for their effective mitigation. A comprehensive analysis of the methodologies used for monitoring biotic and abiotic parameters is beyond the scope of the present paper as they differ substantially, mainly due to the size of the water body (for a detailed review, see [9]). Nevertheless, it is worth mentioning that among the wide range of monitoring techniques and devices used by water authorities and managers of water bodies, fluorescence probes are highly effective because they allow for the real-time continuous monitoring of cyanobacteria and algae. The deployment of such water quality monitoring systems with multi-sensor probes in tandem with fluorescence devices allows one to follow temporal variations to be monitored using the concentration of chlorophyll and accessory pigments as surrogates for phytoplankton, including cyanobacteria in water [10,11]. In addition, very fast progress is being made in implementing remote sensing for cyanoHAB monitoring. It is expected that satellites and drones equipped with multichannel detectors using sophisticated sensors, combined with the development of algorithms and “boots on ground” verifications, will enable the early detection of hot spots of cyanobacteria blooms [12,13,14]. Satellite remote sensing has been widely used to monitor the water quality of inland and coastal waters, and Ho et al. [12] showed an increase in peak summertime bloom intensity in many large lakes worldwide from 1982 to 2012. However, their findings were questioned based on technical and statistical reasons [15]. A more regional approach for the improved and accurate estimation of cyanobacterial pigment concentration in inlands water was demonstrated by Kwon et al. [16], who used drone-based hyperspectral imagery and improved bio-optical algorithms.

### 2.2. Water Shade Management—Nutrient Loads

It is well accepted that phytoplankton will not flourish and form substantial blooms without a proper supply of nutrients. It is not surprising, therefore, that the leading premise/theme/dogma in the field is that eutrophication is a major driving force of the intensification of toxic cyanobacteria blooms, in addition to global warming [8,17,18,19,20]. Contrary to this widely accepted view, the analysis by Ho and co-workers [12] of near-surface phytoplankton blooms from 71 large lakes around the world, covering three decades using Landsat 5 data, showed that exacerbation of bloom conditions in the majority of the lakes did not consistently track with previously hypothesized drivers. In view of the lack of correlation between nutrient loading and bloom intensity, the authors concluded that nutrient reduction targets that are based on historical relationships between bloom severity and nutrient loading may need to be revised in the context of climate change [12,21]. We expect future studies assisted by more satellite and ground confirmations will shed more light on this important aspect.

Nevertheless, with eutrophication being a leading theme, the emphasis is on reducing the nutrients load from the drainage area and thus the nutrients inventory in the water bodies [22]. As tedious, and time-consuming, and resource-consuming as this process may be, it is likely that it may lead to a significant reduction of cyanoHAB events and bloom intensities in the long-run. As the nutrient-bloom congruence has (and still is) been investigated in many studies and summarized in several review articles [6,8,18,23], we do not elaborate on this topic further. However, we draw the attention of the scientific community and water management authorities to the idea that they should anticipate a massive change in phytoplankton diversity along with a reduction in the eutrophication state. As an example, filamentous cyanobacteria capable of N_2_ fixation and cylindrospermopsin (CYN) production may benefit from the declining P and N availability [24,25,26]. This calls for a better understanding of the biological role of cyanobacterial secondary metabolites and specifically those defined as toxins, which is beyond the scope of this review (see [27] on this issue).

### 2.3. Hydrological Manipulation

It is generally assumed that reducing water retention times in a water body to values close to the doubling time of cyanobacteria (up to a few days) may successfully reduce cyanobacterial biomass [28,29]. Earlier studies demonstrated that the flushing of a P-enriched hypolimnion with low phosphorus water could reduce internal P loads and effectively reduce cyanobacterial proliferation [30]. However, it may be difficult to lower the P load using this approach, particularly in large water bodies with seasonal floods and irregular coastlines and many bays, where retention time may not be homogenous. In many cases, retention times are not known but indirectly estimated from an inaccurate water budget and variations in the concentration of a conservative tracer (e.g., chloride ion). The manipulation of residence time (dilution rate) that is suitable to small water bodies, if applied, should consider nutrient loads and directly affect the spatial heterogeneity of phytoplankton biomass [31]. Interestingly, reducing the water retention time (thereby increasing the flashing rate) to control cyanoHABs requires extremely high water inflow rates, whereas flushing out phosphorus can be successfully done for thermally stratified water bodies when fresh water flows via the P-rich bottom layers. Nevertheless, it should be kept in mind that this approach involves the relocation of the phosphorus to a downstream water body, namely potentially moving the problem to a neighbor’s territory [32].

## 3. In-Lake Treatments

The implementation of in-lake treatment is an essential action to mitigate cyanoHABs. The ecosystem response to bloom prevention via the reduction of nutrient loads is a rather slow process. Meanwhile, cyanoHAB events frequently occur and should be treated. Numerous approaches are being applied or proposed to control/mitigate cyanoHABs, from the removal of the cells and limiting the nutrient availability to direct chemical treatments. The advantages and disadvantages of various in-lake treatment approaches are briefly summarized in the specific sections below.

### 3.1. Harvesting of the Floating Cells

**Figure 2:**
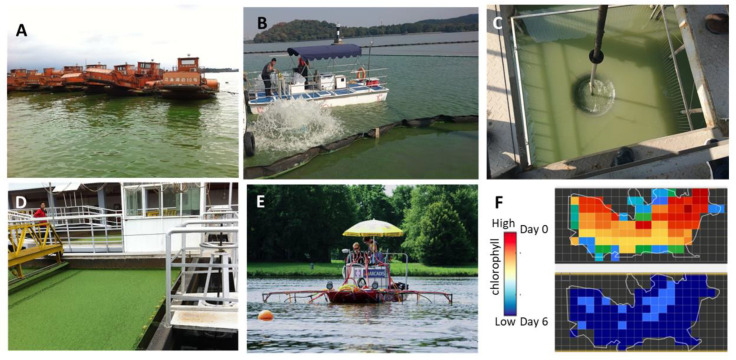
Various techniques used to mitigate and remove cyanoHABs from water bodies. (**A**) A fleet of boats carrying screening devices to remove *Microcystis* colonies (Dian Lake, Dianchi, China). (**B**) A boat carrying and operating ultrasound devices in Tai Lake, China. (**C**) An experimental pit to apply hydrostatic pressure and thereby collapse the gas-vesicles and inhibit colony floatation (taken at Lake Tai, China). (**D**) Flocculation–floatation treatment to remove biomass from *Microcystis*-laden water during a cyanoHAB event in Tai Lake, China. (**E**) The ‘water harrow,’ a device used to disperse hydrogen peroxide into Lake Koetshui ([65], with permission). (**F**) Chlorophyll distribution estimated from satellite spectral images of Lake Nanhu, China, at days 0 and 6 after treatment with an encapsulated floating composition containing copper sulfate (adapted from https://bgtechs.com/, with permission. Accessed date 7 July 2021).

Numerous methods have been developed and examined for the removal of floating toxic cyanobacteria, mostly *Microcystis* sp. by mechanical harvesting (see [33,34,35,36,37,38,39,40] and references therein). Examples from large-scale operations, including biomass micro-screening and flocculation–flotation, are shown in Figure 2. For methodologies where chemically induced aggregations or physical separation are applied, see Section 3.4. The main problems in the biomass harvesting approach is getting rid of the accumulated toxic biomass and avoiding the contamination of underground water with cyanotoxins, a matter of serious concern. It was also proposed that the removal of the cells may reduce the nutrient load [41], but the same problem applies here as well. In addition, in nutrient-rich lakes, the removal of the floating cyanobacteria may simply make room for more cells to flourish.

### 3.2. Nutrient Removal

The reduction of phosphorus (P) concentration in eutrophic lakes, particularly in shallow water bodies, is proposed as an efficient mitigating strategy [42]. Indeed, the application of lanthanum-modified bentonite, commercially known as Phoslock^®^, was able to reduce the P level (sometime transiently) and the bloom intensity in the water column [43,44]. However, reports have also demonstrated potentially serious consequences of the lanthanum level in plants, phytoplankton, zooplankton, and fish, a matter of serious concern [45,46,47,48].

Dredging as a means to remove nutrients and heavy metals from the sediments was proposed and has been applied to small lakes [30,49,50]. If properly performed, it may lower the amount of nutrients in the sediments that may become available in the photic zone for phytoplankton growth. However, given the nature of the involved procedures and their current cost, it may only be applicable to small water bodies. In addition to the removal of the cells from the water column, “Flock and Lock” approaches (see Section 3.5 below) may be an efficient means to lower nutrient availability.

### 3.3. Hydrophysical and Physical Control

#### Buoyancy Regulation

One of the main eco-physiological advantages of cyanobacteria is their ability to modulate/regulate their location in the water column through a balance between the uplift due to the gas vesicles and the ballast, which mainly consists of polyphosphate bodies and photosynthetic products [4,51,52]. The properties of the gas vesicles—in particular, the hydrostatic pressure and ultrasound wave needed to collapse them—differ between species. The collapse of the vesicles following hydrostatic pressure may be used to sediment *Microcystis* sp. cells. In practice, digging holes in the bottom of the water body and pumping the cells downward exposes them to the pressure required to collapse the vesicles and avoid or delay floatation [53,54]. An example from Lake Tai, China, is shown in Figure 2.

Ultrasonic radiation that impairs the stability of the gas vesicles [55,56,57,58,59,60] is also being used to mitigate cyanoHABs (Figure 2). The reader may also refer to various commercial uses (see, for example, lgsonic.com/ultrasonic-algae-control/, Accessed date 7 July 2021). At relatively low frequencies and depending on the power density, microwave radiation affects the growth and metabolism of cyanobacteria [61,62]. Since the sensitivities of the vesicles differ between species, the ultrasonic frequency, radiation durations, and densities must be adopted accordingly. To address the high energy consumption and small effective distance of conventional ultrasonic treatments, a method based on two applications of low-frequency, low-density, and short-duration ultrasonic radiation was proposed [57]. This study also showed that the effective distance of ultrasound decreased with increasing frequency and that the damaged algae cells were able to repair themselves when exposed to low ultrasonic densities [57]. We expect the coverage area to depend on various parameters such as cell density. The impact of the technology on other organisms in the water body, under field conditions, is not clear since other algae and zooplankton may also be susceptible to the treatment, depending on the used frequency and densities [63,64]. Open questions related to both the hydrostatic pressure and ultrasound technologies relate to the ability of the cells to also rebuild their gas vesicles in the dark. This is likely affected by the preceding light intensity and the amount of the accumulated reserve carbohydrates [52]. Naturally, the ability to recover the gas vesicles could severely impact the efficacy of these mitigation approaches.

### 3.4. Chemical Treatments

The development of novel and selective algicides for safe application in various water bodies is essential to mitigate cyanoHABs and preferably assure the persistence and long-term dominance of non-toxic algae following the reduction of the cyanoHAB population. Like other management techniques, algicide must be properly applied to work effectively and to minimize side effects such as the massive release of toxins or adverse effects on non-targeted organisms. These concerns tend to limit algicide applications to special circumstances such as an emergency measures, particularly where alternative drinking-water sources are not available or to restore recreational activities. In most cases, algicides are considered where preventive measures are not feasible or not yet effective [32] or too late to apply.

In general, it is strongly recommended that applications of various algicides should take place at an early stage of bloom development, when cyanobacteria density is low. It may enhance the effectiveness of the treatment and reduce the concentration of toxins that are released from treated biomass. In this context, we propose the concept of “prophylactic treatment,” analogous to a medical approach designed to prevent the occurrence of an adverse event, a disease, or its dissemination. In this concept, repeated applications of low algicide doses may prevent the development of cyanoHABs.

Algicides include a wide range of chemicals that severely affect algae or cyanobacteria (cyanocides) and consequently prevent or mitigate blooms. One example is copper salts, which are frequently applied to control cyanoHABs. Copper is an essential microelement for phytoplankton growth since it participates in numerous oxidative/redox activities (such as cytochrome oxidase and ascorbate peroxidase), as well as in electron transfer from photosystem II to photosystem I in the copper-containing plastocyanin. However, excess copper concentrations inflict severe damage to the cells, similar to other heavy metals. In addition, like Fe^2+^, Cu^2+^ can promote Fenton-like reactions that lead to the formation of hydroxyl radicals, particularly under conditions where H_2_O_2_ is present [65,66,67]. Copper is regarded as the algicide of choice for its effective, relatively safe, and easy application [32]. An example where large-scale treatment with a copper salt effectively cleared a massive cyanoHAB population in Nanhu Lake in China is shown in Figure 2F (adapted from bgtechs.com/, accessed date 7 July 2021). Here, the copper salt was encapsulated within a floating agent (the nature of which was not revealed), leading to the floatation of the algicide and its slow release at the water surface where most of the cyanoHABs were located. Furthermore, the applied material migrates with the wind, as do the cyanobacteria. Regarding its characteristics, the amount of copper salt used was 1–4 g/m^2^ (depending on local cyanobacteria abundance). Nevertheless, the application of copper sulfate has been restricted due to its tendency to accumulate in lake sediments and be released back to the open water, affecting sensitive organisms including fish and thus becoming an “ecological finger print.” Repetitive use may also induce the emergence of copper-resistant cyanobacteria. Chelated copper compounds are replacing copper sulfate treatments as a safer and more effective substitute. However, despite its effectiveness and the very low concentration used when encapsulated, the growing public awareness of environmental issues is driving the current trend to reduce copper applications in inland waters.

Unlike copper, aluminum is not considered an essential element for the growth of cyanobacteria, but its additions stimulated the growth of the marine cyanobacterium *Synechococcus* WH7803 and the N_2_-fixing *Trichodesmium* sp. [68]. Aluminum salts are being used to mitigate toxic blooms, mostly but not only by coagulation (see below). Apparently, aluminum sulfate (alum) treatment also reduces P availability and decreases algal bloom frequency and density. As an example, a 70% reduction of chlorophyll-a content was obtained following an aluminum sulfate treatment (a dose as high as 84 g/m^2^ was applied) to a hypereutrophic lake. This treatment also led to a 95% reduction in total phosphate concentration, likely due to the formation of insoluble aluminum phosphate salt that may have led to P limitation [69].

#### 3.4.1. Oxidative Stress Based Treatments

Oxidative stress is becoming a major approach to mitigate toxic blooms. In general, cyanobacteria are far more sensitive to oxidative stress, such as H_2_O_2_ applications, than many eukaryotic phytoplankton [65,70,71,72,73,74,75,76,77], most likely because they display a lower ability to decompose H_2_O_2_ than eukaryotic algae [78,79]. The evolutionary reasons for this observation are not clear but may be related to the fact that, in cyanobacteria, light-dependent O_2_ uptake in the photosynthetic electron transport route is mediated via flavodiiron (known as FLV proteins) that release water [80]. This is in contrast to the formation of reactive O_2_ species (ROS) during O_2_ uptake by the thylakoid-located photosynthetic machinery, the Mehler reaction, in algae and plants [81]. Another source of ROS is the oxygenase activity of the universal CO_2_-fixing enzyme—ribulose bisphosphate carboxylase/oxygenase (RubisCO). The metabolism of the produced 2-phosphoglycolate releases H_2_O_2_ in the peroxisomes [82]. The efficient CO_2_-concentrating mechanism in cyanobacteria [83] lowers the oxygenase activity of RubisCO and hence the formation of H_2_O_2_. The production of ROS in the photosynthetic machinery may have been the evolutionary driving force that led to a greater expression of multiple mechanisms to detoxify ROS in algae and plants.

Hydrogen peroxide is considered the primary reagent to impose oxidative stress under environmental conditions and may be provided as a liquid solution [65,84]. Other alternatives have been proposed and evaluated, including sodium per-carbonate (SPC) that releases H_2_O_2_ and generates reactive oxygen species, superoxide radicals, carbonate radical anions, and hydroxyl radicals [77,85]. Similarly, metallic peroxide granules such as calcium peroxide (CaP) and MgO_2_ [86,87] and peracetic acid [88] have also been considered. Experiments where various H_2_O_2_ concentrations were applied to assess the effective concentration showed the differential sensitivity of various toxic cyanobacteria to H_2_O_2_. *Planktothrix* showed the highest sensitivity, *Microcystis* was moderately sensitive, and *Cylindrospermopsis* was the most resistant [75,78,89]. Another set of experiments showed the following order of sensitivities: *Pseudanabaena limnetica* > *Raphidiopsis curvata* > *Cylindrospermopsis raciborskii* [77]. Noticeably, the sensitivity to H_2_O_2_ is strongly affected by biotic and abiotic conditions such as nutrient status [76], prior exposure to ROS [90], and the presence of other organisms that may possess a significant H_2_O_2_ detoxification capability [78]. The cell density and phase of growth are also important biotic factors that affect sensitivity to H_2_O_2_ applications [90]. Stationary cultures have shown a much higher ability to decompose H_2_O_2_ than younger cultures, reflecting the induction of genes engaged in antioxidant activities in the former that already experienced an earlier stress [90].

The “ecological finger print” of H_2_O_2_ treatments across the concentrations used has been examined in laboratory and small-scale outdoor experiments. These have included the impact on bacteria, zooplankton, and phytoplankton, particularly under H_2_O_2_ concentration above 4 mg/L, which is the threshold lethal dose for *Microcystis* sp. [65,73,75,91,92,93,94]. Though there has been some variability between the reports, likely reflecting local biotic and abiotic conditions, the overall conclusion was that the H_2_O_2_ dose required to significantly lower the population of toxic cyanobacteria also affects other organisms (see [84] in this special issue). As an example, in a large experiment performed on four small lakes in New York State, Lusty and coauthors [75] found that the bacteria phyla *Actinobacteria*, cyanobacteria, *Planctomycetes*, and *Verrucomicrobia* were most negatively impacted by H_2_O_2_, with *Actinobacteria* being the most sensitive. On the other hand, there are indications that the suppression of the cyanobacteria population enables the recovery of eukaryote algae. One example is the study of Fan and co-authors [38], who found a dynamic change in the phytoplankton population composition and dynamics, notably a decline in the abundance of *Microcystis* but a rise in chlorophytes. Naturally, a significant impact on other organisms raises doubts about the efficacy of H_2_O_2_ treatment as an “ecologically friendly” approach. Would it be possible to reset the phytoplankton population composition to the pre-toxic cyanobacteria era?

Interestingly, the *Microcystis* sp. strain MGK was far more sensitive to two consecutive low concentration H_2_O_2_ doses (lower than the lethal dose) given within 1–6 h apart than to a single dose where the H_2_O_2_ concentration was higher than in the combined two treatments [90]. These data recalled a similar case in the green algae *Chlamydomonas reinhardtii* where two consecutive treatments of low H_2_O_2_ concentration (much lower than the lethal dose) induced a program cell death (PCD)-like process involving the executioner caspase proteins [94]. The first H_2_O_2_ application upregulates genes encoding for enzymes engaged in H_2_O_2_ decomposition such as ascorbate peroxidase (APX). The first product of APX activity, dehydroascorbate (DHA), is produced following the second H_2_O_2_ treatment, serves as a surveillance molecule reporting to the cell that it already experienced oxidative stress, and thus activates the PCD-like process [95]. The application of DHA to cells that were not exposed to oxidative stress was found to activate the PCD-like process. The mechanism of PCD activation by the DHA level is unknown, as is the evolutionary driver that altruistically kills the cells. A likely possibility is that it aims to enhance the population fitness by eliminating cells that were damaged by oxidative stress. It has not been fully established whether this is also the case in cyanobacteria, including *Microcystis* sp., but there is an increasing body of evidence that oxidative stress induces a PCD-like process in cyanobacteria and that some of the components engaged in green algae are also present here [96,97,98,99,100,101,102,103,104,105].

**Figure 3:**
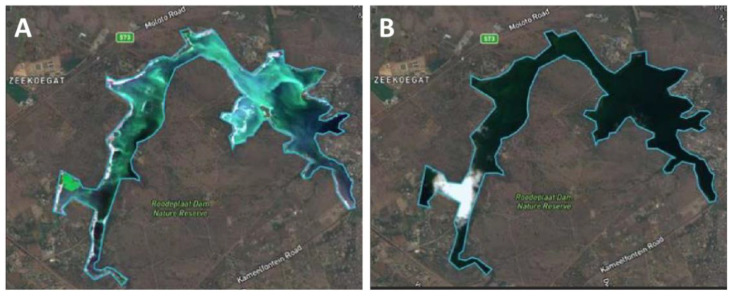
A satellite picture (unprocessed) of Roodeplaat dam area in South Africa (**A**) before and (**B**) 10 days after treatment with a floating composition that releases H_2_O_2_ (adapted from https://bgtechs.com/, with permission, accessed date 7 July 2021). A cloud is present in the left side of panel B.

Large-scale treatments with positively buoyant, encapsulated SPC have been used effectively to treat massive cyanoHABs (see an example in Figure 3. Image was adapted from bgtechs.com/, accessed date 7 July 2021). This application isbased on the slow release of H_2_O_2_ at the water surface where most of the cyanoHABs are located. The applied encapsulated SPC migrates with the wind to meet the accumulated scum of cyanobacteria. Importantly, the concentration of H_2_O_2_ applied over the entire water body is significantly lower than the lethal dose determined in laboratory and small-scale lake experiments, such as in [65,75]. This raises the possibility/speculation, yet to be experimentally verified, that long exposure to oxidative stress (due to the slow release of H_2_O_2_) induces a PCD-like process that propagates within the *Microcystis* sp. population. However, as peroxides are applied to mitigate cyanoHABs, their effectivity and selectivity should be considered in the whole ecosystem context. For example, the sensitivity of the microbial community and other higher trophic levels organisms should be examined.

#### 3.4.2. Other Algicides and Cyanocides

In a comprehensive overview, Matthijs and colleagues [86] evaluated common herbicides such as copper sulfate, diuron, and endothall to control cyanoHABs. They recommended replacing these algicides with newly emerging compounds that show better specificity for cyanobacteria and are biodegradable or transformable into non-toxic products after application. Many products isolated from plants and microorganisms, from barley straw to biologically active compounds and their synthetic homologues, were identified as potent cyanocidal (causing cell death) or cyanostatical (causing the inhibition of cell proliferation) agents [106]. In addition, synthetic surfactants such as quaternary ammonium compounds (QACs), which are commonly used in the food industry, have been reported as algicides and active bacteriocides [107,108], and they were recently evaluated as effective cyanocides [109]. Alkyl trimethylammonium compounds (ATMAs), a class of QAC cationic surfactants, inhibit photosynthesis in cyanobacteria and algae with some selectivity toward cyanobacteria [109]. QACs have represented effective classes of disinfectants for nearly a century [110] and have a detergent-like mechanism of action against microbial life. Electrostatic interactions between the positively charged head and the negatively charged bacterial cellular membrane are followed by the permeation of the side chains into the intramembrane region, ultimately leading to the leakage of cytoplasmic material and cellular lysis.

Clays modified with hexadecyltrimethylammonium bromide (CTAB) were implemented to clean blooms of *Microcystis* in Lake Tai, China [111]. Similarly, granulated composites of bentonite with micelles of octadecyltrimethylammonium (ODTMA) bromides, as well ODTMA ions, had a deleterious effect on cyanobacteria cells [109,112]. The application of disinfectants such as ODTMA or other QACs may present potential biocidal effects on non-target aquatic organisms, protozoa, crustacean, fish, and microbial community, although cyanobacteria are more sensitive to the presence of QACs than fish and crustaceans [109].

### 3.5. Removal of CyanoHAB Biomass by Chemical Treatment

The agglomeration (coagulation/flocculation) phase is one of the most important steps in algal removal during water treatment. This process, which is induced by the addition of chemicals, destabilizes particles in solution by neutralizing their surface charge. Coagulation and flocculation principles have been applied for the in-lake reduction of cyanoHABs, with aluminum or iron-based compounds as the most widely used chemicals. Precipitating the cyanobacteria cells using a low-dose coagulant, such as polyaluminum chloride, transiently reduced the surface cyanobacterial chlorophyll-a by 90%, but floatation quickly recovered. Since the effectiveness varies with the nature of the dominated species, it was proposed that the application should be adopted to the specific water body and applied with additional means to remove P [43]. Recently, Lürling et al. [113] reported the use of mineral/metal-based, natural, organic, and synthetic coagulants combined with a ballast to enhance the in-lake sedimentation of the cyanobacteria aggregates, generally termed the “Floc and Sink” approach. The application of synthetic and preferably natural polymers, such as chitosan, for cyanoHAB flocculation was also investigated, including the effect of extracellular organic matter on flocculation efficiency [35]. Magnetic nanoparticles such as polyethylenimine-coated iron oxide were also applied to sediment *M. aeruginosa* [39], and guidance on cyanobacteria harvesting using magnetic separation technology under different environmental conditions was given [40]. An alternative approach proposes the use of local clay-enriched soil modified with cationic starch [114], chitosan or proteins [115,116]. Positively charged proteins neutralize the negative charge on the algal cells’ surfaces and destabilize them to form small flocs. Chitosan, with its long polymer chain, links and bridges from the small flocs into large ones. Together with soil particles, the flock settling is accelerated and a high removal efficiency is achieved in a short time. Nevertheless, cell damage and the potential leakage of toxins should be considered under these circumstances. In addition, the effect of the added chemicals on the microbial community and other organisms of higher trophic levels should be examined.

### 3.6. Approaches toward Biological Treatments

A range of techniques aimed at the manipulation of phytoplankton community composition and growth, often termed “biomanipulation or top–down,” aim at stimulating the growth of zooplankton that graze on phytoplankton. Another approach aims at stimulating the growth of submerged aquatic plants (“macrophytes”) or seeds that can compete with phytoplankton for nutrients and provide refuges for zooplankton. The reader is referred to the work of Burch et al. [32] (and references therein), who addressed these measures, described a few successful cases, and discussed the environmental conditions that might support a successful operation.

**Figure 4:**
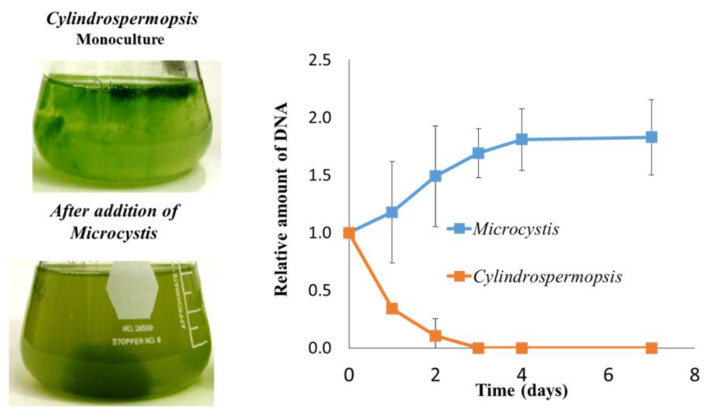
*Microcystis–Cylindrospermopsis* interaction. A monoculture of non-toxic *Cylindrospermopsis raciborskii*, mostly floating filaments (top Erlenmeyer flask), clustered together and sank to the bottom after the addition of *M. aeruginosa* sp. MGK cells (bottom Erlenmeyer flask). The right panel shows the changing level of specific DNA from each of the organism gauges via 16S rDNA analyses.

Here, we highlight an emerging venue based on intra- and inter-species communication/competition/allopathic interactions that take place between toxic cyanobacteria, non-toxic cyanobacteria (mainly *Microcystis* sp.), and other organisms, mainly (but not only) green algae, that may ultimately lead to the development of mitigation protocols (see [66,75,89,117,118,119,120,121,122,123,124,125,126,127,128,129,130,131,132,133,134,135,136,137,138,139,140,141,142,143,144,145] and references therein). An emerging example is the reduction of cyanoHAB populations that enables the persistence dominance of various non-toxic algae. Studies on the interactions between various toxic and non-toxic *Microcystis* cells in cultures and within their floating colonies, as well as with other cyanobacteria [146,147,148,149,150,151,152], suggested that in addition to the known toxins, other secondary metabolites are involved in the “languages spoken in the water bodies” [117,153]. One example is the interaction between *Microcystis* and *Cylindrospermopsis*. The addition of *Microcystis* cells led to the sinking of an earlier floating *Cylindrospermopsis* culture to the bottom of the flask and a decline in its specific DNA level (Figure 4). With the exception of the toxins, the nature of the active components that may act as allelochemicals were not revealed in most cases [24,154,155,156]. This calls for the identification of their nature via intensive chemotyping, paving the way to develop biological approaches towards the mitigation of cyanoHABs.

The use of bacteria to control toxic blooms was proposed [157]. One example is *Aeromonas veronii*, which is often found in association with *Microcystis* colonies [158,159,160]. Spent media from *A. veronii* isolated from *Microcystis* sp. colonies inhibited the growth of *M. aeruginosa* sp. MGK. The inhibition was much stronger when the growth medium of *A. veronii* contained spent media from MGK, suggesting that *A. veronii* recognized the *Microcystis* presence and produced secondary metabolites that inhibited its growth. Fractionations of the *Aeromonas* spent media identified lumichrome as the active component; its application at concentrations as low as 4 nM severely inhibited *Microcystis* sp. MGK growth [159].

An interesting interspecies interaction between *M. aeruginosa* and the green alga *Scenedesmus quadricauda* was revealed in a recent study that focused on the impact of N-phenyl-2-naphthylamine (PNA), a root exudate produced by the invasive floating macrophyte *Eichhornia crassipes* (water hyacinth) [138]. PNA differentially inhibits both organisms, but the impact on S. *quadricauda* was found to be much stronger when *Microcystis* was present [138]. Similarly, when both organisms were treated with the antibiotic norfloxacin, the inhibition of *S*. *quadricauda* was much stronger when *Microcystis* was also present [135]. These and many other reports suggest that exposure of *Microcystis* to stress enhances the intensity of interspecies interactions. This may be applied in future studies to identify the allelochemicals involved in order to use them to control toxic cyanoHABs.

## 4. Drinking/Potable Water Treatment—Removal of Cyanobacteria and Their Toxins

### 4.1. General Considerations

CyanoHABs in freshwater ecosystems pose a significant challenge to water authorities because the implementation of special treatments is required in order to eliminate residual toxic cells and to remove soluble toxins from drinking water. The World Health Organization (WHO) proposed guidelines for cyanotoxins in drinking water [161,162,163,164], and many local and national water authorities endorsed these guidelines. The success of a comprehensive plan for cyanotoxin removal to meet these guidelines calls for a broad understanding of the physico-chemical properties of cyanotoxins, their biological nature, their origin (intracellular or extracellular), and their temporal and spatial distribution in water sources.

Freshwater sources are commonly used to provide drinking water, and water treatment facilities are designed and operated to bring the raw water to the required quality of drinking water. In the case of cyanoHABs in source water, a flexible inlet site may reduce contamination because blooms tend to unevenly disperse in the water column and lake area [165]. Nevertheless, the elimination of cyanotoxins from inlet water, both soluble and particulate, requires close monitoring over the time and space of bloom development and, in extreme cases, may necessitate the application of urgent protocols [49]. It is clear that the application of physico-chemical or physical means (e.g., algicides, coagulation/flocculation, and hydrodynamic cavitation) to control in-lake cyanoHABs should be done cautiously, as the death and lysis of toxin-producing cyanobacteria release toxins into ambient waters. This can lead to higher concentrations of cyanotoxins in the raw water, affect the quality of drinking and irrigation water, and restrict the use of the water for recreational activities [166].

### 4.2. Water Intake

Early warning systems to identify cyanoHAB development are considered an important source of information for drinking-water treatment plants (DWTPs) that take raw water from above-ground sources. The information can be acquired from real-time monitoring systems located at the vicinities of pumping stations and from hyperspectral or multispectral devices mounted on satellites or drones [16,167,168]. Satellites provide large-scale areal data, but this source is limited by the low frequency of data acquisition. This limitation can be overcome by the operation of drones from a land base or nearby elevated site equipped with a multispectral camera covering the DWTP intake site [16]. The uneven vertical distribution of the cyanobacteria population in the water column presents an additional complexity that calls for the development of advanced methodologies for in situ real-time monitoring capacity [169]. The gathered information can be used as an input for forecasting models to predict the development of cyanobacteria and their toxicity in a DWTP inlet [170]. Several management strategies may be taken by drinking-water utilities to minimize the intake of raw water enriched with cyanobacteria and their toxins: (1) use an alternate supply, (2) adjust intake depth and time, and (3) treat the intake water. It is uncommon for drinking-water utilities to have access to more than a single source of water, so the simplest strategy to change sources in the case of cyanoHABs is irrelevant [6,171]. Adjusting intake depth as an alternative may be effective in cases where the toxic biomass concentrates in relatively narrow water layer and where the intake depth is flexible. Some cyanoHABs occur at limited depths in the water column and are characterized by a diurnal pattern of vertical migration, which is affected by the biological regulation of cell/colony buoyancy and changes in turbulence and thermal stratification under different environmental factors and various hydrodynamic conditions [165]. Therefore, attempts to draw water from different depths or at specific times to avoid the pumping of contaminated water and cells into a treatment plant is recommended [171]. The effectiveness of this recommendation can be evaluated by the real-time monitoring of the source water at specific depths or at the pumping station by fluorescence [10], in addition to the intermittent analysis of cyanotoxins using rapid-response ELISA kits [172].

Optimizing the treatment of intake water laden with cyanoHABs is very important, since many cyanotoxin (microcystin variants, anatoxin-a, and saxitoxins) are found intracellularly. Cylindrospermopsin, however, is naturally released from the cyanobacterial cells, and the extracellular CYL may account for 50% of the total [173,174]. When a bloom declines, cell growth slows, the toxic population senesce and degrade, and intracellular toxins are released to the surrounding water [175].

### 4.3. On-Line Pretreatment

Oxidants are often added at the DWTP intake. As discussed in Section 3.4, oxidative treatments are aimed to reduce taste and odor compounds, depress biological growth on the intake pipe (zebra mussels, biofilm, and algae), reduce the production of disinfection by-products, and stimulate coagulation [171,176]. Regarding cyanoHAB contamination, the addition of an oxidant at the intake may lyse cells and consequently increase the concentration of dissolved organic matter (DOM), including soluble toxins. In most cases, the concentration of the applied oxidant is not sufficient to oxidize the pool of dissolved toxins. Chlorination at the intake is undesired due to the formation of by-products and the risk of lysing cyanobacteria cells [177,178]. Similarly, pretreatment by ozonation has been found to impose cell wall damage and the release of microcystins and other cellular compounds [179,180]. However, the application of ozone may efficiently remove MCs but affect subsequent coagulation steps [181]. Pretreatment with potassium permanganate was reported to improve coagulation with minimal effects on cell integrity and the release of toxins [180,182].

### 4.4. Removal of Suspended Matter

The conventional DWTP consisted of the aggregation of suspended matter via coagulation/flocculation protocols followed by aggregate removal using sedimentation or floatation techniques. The agglomeration (coagulation/flocculation) phase is one of the most important steps in cyanobacteria removal. In this process, chemicals are added to destabilize suspended cells and colonies by neutralizing their surface charge. Coagulants used for the removal of cyanobacteria include multiple positive charge chemicals such as ferric sulfate, polyferric sulfate, and alum poly-aluminum chloride [183,184,185,186]. Aluminum- and iron-based compounds have been the most widely used chemicals for the successful coagulation and removal of cyanobacteria biomass, with minor releases of toxins [184,185], although other studies have demonstrated that the application of ferric or aluminum coagulation could induce toxin release from *Microcystis* and *Planktothrix* toxic species [187]. Furthermore, recent studies in microcosm experiments demonstrated extended cell damage and toxin release from *Microcystis* cells upon the application of alum [188].

Organic polyelectrolytes are frequently used in DWTP for coagulation and flocculation, as well as in the sludge dewatering step [189]. Polymers lower the coagulant dose requirements, produce a smaller volume of sludge, and decrease the ionic load and level of aluminum in the treated water. The application of organic polyelectrolytes in water treatments were reviewed in [189], with emphasis on the mechanisms of coagulation and flocculation with polymers commonly used in DWTP. The most effective organic polymers for the removal of cyanobacteria are cationic polymers that usually, but not always, possess quaternary ammonium groups that have a positive charge regardless of pH. Some natural products or their derivatives, such as chitosan, are also employed in water treatment. These cationic polymers have antibacterial activity associated with cell disintegration and the leakage of cyanobacterial toxins [112,190,191]. Applications of advanced nano-composites (comprising modified clay) for environmental remediation were reviewed in [192], whereas their potential use for the flocculation of cyanobacteria and their toxins recently emerged [112,193].

Considering the fact that soluble toxins are hardly removed by coagulation, the addition of an adsorbent such as powdered activated carbon (PAC) to coagulation was evaluated. In that process, PAC was added to the source water to maximize contact time followed by the removal of the suspended particulate material during sedimentation [178].

The removal of cyanobacteria biomass is commonly achieved by sedimentation or dissolved air flotation (DAF), with the latter being found more effective because the floating capacity of aggregated colonies and cells improves that process [194]. In DAF, fine air micro-bubbles are introduced to the liquid phase. The micro-bubbles attach to and are trapped in the aggregated particles that become lighter and then float on the water surface. An efficient DAF process requires particle coagulation that destabilizes them by neutralizing their negative charge and changes their hydrophilicity [195,196]. The concentrated cyanobacterial biomass in the form of dense sludge needs special attention because cell lysis is accompanied by the release of intracellular toxins prior and during sludge storage and treatment [194]. Therefore, the disposal of sludge containing cyanotoxins must confirm the absence of residual toxicity and comply with regional legislation concerning toxic wastes.

Traditional DWTPs are based on conventional processes, e.g., flocculation, sedimentation, and filtration, where the filtration step is based on slow sand filtration or filtration via rapid sand filters or higher-rate filters (i.e., dual-media and multimedia filters). Sand filtration involves the passage of water by gravity through a filter of granular material to remove any remaining particulates in the water following sedimentation. The remaining cyanobacteria cells are effectively removed during filtration. Practically, no removal of soluble cyanotoxins occurs during rapid sand filtration via physical and/or chemical mechanisms, but a microbial community capable of toxin biodegradation may develop on the filters [178]. The operation of a conventional DWTP during a cyanoHAB event requires the frequent backwash of filters because the retained cyanobacteria cells may release intracellular toxins to the main filtrated water stream.

### 4.5. Membrane Filtration

The integration of membrane processes such as microfiltration (MF) and ultrafiltration (UF) in DWTPs is becoming increasingly widespread [183]. These processes are well-suited to remove cyanobacteria, filaments, colonies, and single cells by physical separation, thereby eliminating most of the intracellular toxins. MF and UF processes are commonly used in small-to-medium-size water treatment facilities and substitute coagulation–rapid sand filtration processes [171]. The amount of toxin released due to disintegration of cyanobacterial cells by sheer forces applied during the UF process should be further evaluated. Dixon et al. [183] showed the leakage of cyanotoxins during ultrafiltration experiments. Molecular mass cut-off and intermolecular, van der Waals, dipole–dipole, hydrogen bonding, and electrostatic interactions all play important roles in determining the acceptance/rejection criteria of each membrane toward cyanotoxins. Therefore, fundamental research is needed to reveal the mechanism of cyanotoxin rejection by membranes.

### 4.6. Removal and Degradation of Soluble Cyanotoxins

Despite the gross removal of cyanobacteria cells, filaments, colonies, and subsequent intracellular toxins following the conventional steps of DWTP, extracellular toxins may persist and pose a substantial public health risk with respect to acute and chronic exposure. Various technologies have been explored for the removal of soluble cyanotoxins. Here, we briefly describe emerging techniques, some of which are currently in use for water treatment.

#### 4.6.1. Toxin Adsorption

Adsorbing agents are commonly used for the removal of soluble trace contaminants such as cyanotoxins and are selected on the basis of their affinity to the target contaminants. Here, we review the use of activated carbon and other newly-emerging adsorbing materials for the removal of cyanotoxins from water.

Activated carbon has a large specific surface area (400–1500 m^2^/g) that provides numerous adsorption sites for removing organic contaminants. Two types of activated carbon are used in the DWTP: powdered activated carbon is generally used for temporary treatment, as was mentioned earlier, and granular activated carbon (GAC) is used in fixed beds to adsorb cyanotoxins efficiently [197]. Different precursors and processes used to activate the carbon result in variable adsorptive capabilities and selectivities for specific cyanotoxins. For example, mesoporous activated carbon (pore size: 2–50 nm) is more effective in adsorbing microcystins than macroporous or microporous activated carbon, whereas saxitoxin removal is more efficient in micro-pore GAC due to its smaller molecular mass [171]. Other chemical properties of cyanotoxins such as hydrophobicity and functional groups affect the adsorption efficiency by GAC. For example, the order of removal efficiency for MCs is MC-RR > MC-YR > MC-LR > MC-LA [198,199]. In addition, the efficiency of a particular type of activated carbon for a certain toxin depends not only on matrix properties (number and size of the adsorption sites or pores) but also on other DOC components in the treated water that compete for adsorption and may reduce the removal of cyanotoxins [178].

GAC filtration systems are operated at relatively slow filtration rates and extended run times. Under such conditions, biofilm that can negatively affect the adsorption capacity of the filter on the one hand but may enhance biodegradation of cyanotoxins on the other hand is formed [200]. This effective biofilm has attracted attention and led to the isolation of microcystin-degrading bacteria and elucidation of the biochemical pathway associated with the degradation of microcystin encoded by the mlrABCD (*mlr*) cassette [201].

Granulated composites of bentonite with micelles of ODTMA were reported as an efficient matrix that rapidly and reliably removes the cells of cyanobacteria and cyanotoxins from laboratory cultures and lake water [112]. The capacity of ODTMA nano-composites to remove microcystins (MCs) from water to below 1 µg/L via filtration was further demonstrated with a high capacity toward MC-LR and other MC congeners (MC-WR, MC-3aspWR, and MC-YR) but a lower affinity to more positively charged MC congeners (MC-RR and MC-3aspRR). Filtration results were simulated with a filtration model that considers the convection and adsorption/desorption of one to several toxins. This further supports the possibility that granulated nano-composites of ODTMA-bentonite can be applied for the removal of microcystins from drinking water [199].

#### 4.6.2. Chemical Processes and Advanced Oxidation

Traditional disinfecting oxidative processes showing variable efficiency in the removal of dissolved cyanotoxins have been reported. The stability of cyanotoxins in these treatments has varied due to their molecular structure and the nature of the applied oxidant. Microcystins have three general areas subject to oxidation: the conjugated double bond in the Adda moiety, the single double bond in the Mdha moiety, and the side chain of the variant amino acids, mainly arginine and tyrosine amino acid [171]. The susceptibility of individual microcystin congeners to chlorination was found to be MC-YR > MC-RR > MC-LR > MC-LA [202]. Oxidation by chlorine [203] or ozone [204] can be effective in degrading dissolved CYN under conditions normally applied for the optimal disinfection of drinking water. However, the type and concentration of organic substance, as well as the pH, strongly affect the amount of disinfectant needed. Other disinfectants such as chloramine and chlorine dioxide are ineffective for CYN decomposition [204]. Permanganate can effectively oxidize ANTX and microcystins, while ozone is capable of oxidizing all three toxins (microcystin, CYN, and ANTX) at a high rate. The formation of trihalomethanes (THMs) in treated water may restrict the application of sufficiently high-chlorine doses.

An alternative methodology for the degradation of cyanotoxins is based on advanced oxidation processes (AOPs) that efficiently degrade recalcitrant organic compounds. In AOPs, hydroxyl radical (∙OH) and other highly reactive radicals are formed in situ and chemically attack and oxidize cyanotoxins. Though ozonation is widely employed in drinking-water treatment, ozone itself can be used as an AOP precursor in combination with other oxidants, UV light, or catalysts. This combination increases the formation of ·OH and further enhances the degradation of microcystins and anatoxins [205]. Similarly, the degradation of these toxins was also found to increase when Fe^2+^ was combined with O_3_. The photocatalyst TiO_2_ can also be used as a substitute of Fe^2+^. Cylindrospermopsin degradation improved when ozone was combined with TiO_2_ due to increased O_3_ decomposition to ∙OH and CYN adsorption to the catalyst [205].

In a recent review, Zhang et al. [206] discussed in detail the application of AOPs for the treatment of cyanotoxins and cyanobacteria. They concluded that AOPs had great potential for the treatment of cyanotoxins and cyanobacteria-contaminated waters, but they indicated that further work is required to establish the practical application for AOPs for the treatment of cyanotoxins and cyanobacteria.

## 5. Concluding Remarks and Perspectives

CyanoHABs constitute an increasing threat to aquatic ecosystems and jeopardize public water supplies around the world. In the long run, the mitigation of cyanoHABs calls for reducing nutrient loads and manipulating environmental factors known to promote cyanoHABs. Hopefully, watershed management efforts will lead to a significant reduction of cyanoHAB events and bloom intensities. Ecosystem-scale mitigation strategies focusing on the reduction of nitrogen and phosphorus inputs are essential and should to be adjusted with changing climatic conditions. While taking the necessary steps to decrease nutrient loads, we must bear in mind the likely effects on the community structure due to biological features of certain cyanobacteria such as the ability to fix atmospheric N_2_, cellular buoyancy, and effective recruitment of P. In the meantime, innovative solutions should be developed and applied to combat cyanoHAB events via the implementation of in situ scientifically proven measures to minimize cyanobacterial biomass and cyanotoxins, as well as to support the domination of other non-toxic algae species. Resetting the phytoplankton composition to the pre-toxic domination era may require an in-depth chemotyping of the produced secondary metabolites and a better understanding of their role in the interspecies interactions that play important parts in biodiversity and its dynamics.

In-lake chemical treatments based on the application of peroxides appear to comprise the most efficient approach that targets the differential sensitivity of cyanobacteria while leaving no chemical residues, thus minimizing effects on the entire ecosystem. The optimal formulation of an oxidation agent and targeting of the active reagent toward the blooming population, either to a scum floating at the water surface or to a metalimnetic stratum, may improve management efforts and reduce oxidant loads. Furthermore, in sites with perennial events of cyanoHABs, a prophylactic treatment using a relatively low concentration of peroxide agents is strongly recommended at an early stage of bloom development, thus restricting the appearance of the cyanobacteria population and supporting the establishment of competing eukaryotic algae. Novel and selective techniques for safe application in lakes and water reservoirs are emerging and are further anticipated in the near future. Such techniques should be timely and properly applied to effectively work to control cyanoHABs and to prevent undesired side-effects such as the massive release of toxins.

An additional important advantage of in-lake prophylactic treatment stems from the relatively minor release of toxic compounds from the pre-bloom toxic cyanobacterial biomass. This stands in contrast to the application of many management techniques during massive blooms, where intracellular substances including toxins are released from both lysing and intact cells, thus strongly affecting cell physiology [27], as well as on other trophic levels. In some cases, the released secondary metabolites may trickle to the groundwater and create a secondary contaminated site. These concerns may limit the use of such raw water as a source for drinking water.

While in-lake treatment serves as a first barrier to eliminate cyanotoxin threats in drinking water, DWTPs that pump water from sites of potential cyanoHABs should adjust their treatment capabilities to minimize risks from toxic cyanobacteria and their metabolites while complying with health regulations. Conventional processes employed for water treatment inefficiently remove soluble toxins and, in some cases, increase their concentration due to the disintegration of toxic cells. The currently developed methodologies for efficient removal of the toxins rests on adsorption of cyanotoxins onto activated carbon, either granulated or powder. Emerging nanotechnology techniques implemented for cyanotoxin adsorption or improved oxidation processes have been proven to be efficient, but they certainly need further examination for large-scale conditions. It is important to note that in many sites, such advanced technology may only be needed for a limited time during cyanoHAB events, and capital investment and operational costs may restrict their availability.

Recruiting microbes capable of outcompeting toxic strains or degrading cyanotoxins is an attractive track that, so far, has only been examined under laboratory conditions. A consortium of various microorganisms specialized in the degradation of different cyanotoxins can be naturally established on various matrices, but the stability of such consortia depends on continuous exposure to the toxins and relatively stable environmental conditions. Future research in this area could improve our understanding of the biodegradation pathways across different cyanobacterial toxins and lead to the design of optimal strategies for toxin removal in drinking-water facilities.

## Data Availability

Not applicable.
